# ParticipACTION: Awareness of the participACTION campaign among Canadian adults - Examining the knowledge gap hypothesis and a hierarchy-of-effects model

**DOI:** 10.1186/1479-5868-6-85

**Published:** 2009-12-09

**Authors:** John C Spence, Lawrence R Brawley, Cora Lynn Craig, Ronald C Plotnikoff, Mark S Tremblay, Adrian Bauman, Guy EJ Faulkner, Karen Chad, Marianne I Clark

**Affiliations:** 1 Sedentary Living Lab, Faculty of Physical Education and Recreation, E-488 Van Vliet, University of Alberta, Edmonton, AB, T6G 2H9, Canada; 2 College of Kinesiology, PAC 300, 87 Campus Drive, University of Saskatchewan, Saskatoon, SK, S7N 5B2, Canada; 3 Canadian Fitness and Lifestyle Research Institute, 201-185 Somerset Street West, Ottawa, ON, K2P 0J2, Canada; 4 School of Public Health, K25 - Medical Foundation Building, The University of Sydney, Sydney NSW 2006, Australia; 5 School of Public Health and Faculty of Physical Education and Recreation, E-488 Van Vliet Centre, University of Alberta, Edmonton, AB T6G 2H9, Canada; 6 Healthy Active Living and Obesity Research Group, Children's Hospital of Eastern Ontario Research Institute, 401 Smyth Road, Ottawa, ON, K1H 8L1, Canada; 7 University of Toronto, Faculty of Physical Education and Health, 55 Harbord Street, Toronto, ON, M5S 2W6, Canada; 8 College of Kinesiology, PAC 300, 87 Campus Drive, University of Saskatchewan, Saskatoon, SK, S7N 5B2, Canada; 9 Sedentary Living Lab, Faculty of Physical Education and Recreation, E-488 Van Vliet, University of Alberta, Edmonton, AB, T6G 2H9, Canada

## Abstract

**Background:**

ParticipACTION was a pervasive communication campaign that promoted physical activity in the Canadian population for three decades. According to McGuire's hierarchy-of-effects model (HOEM), this campaign should influence physical activity through intermediate mediators such as beliefs and intention. Also, when such media campaigns occur, knowledge gaps often develop within the population about the messages being conveyed. The purposes of this study were to (a) determine the current awareness of ParticipACTION campaigns among Canadians; (b) confirm if awareness of the ParticipACTION initiative varied as a function of levels of education and household income; and, (c) to examine whether awareness of ParticipACTION was associated with physical activity related beliefs, intentions, and leisure-time physical activity (LTPA) as suggested by the HOEM. Specifically, we tested a model including awareness of ParticipACTION (unprompted, prompted), outcome expectations, self-efficacy, intention, and physical activity status.

**Methods:**

A population-based survey was conducted on 4,650 Canadians over a period of 6 months from August, 2007 to February, 2008 (response rate = 49%). The survey consisted of a set of additional questions on the 2007 Physical Activity Monitor (PAM). Our module on the PAM included questions related to awareness and knowledge of ParticipACTION. Weighted logistic models were constructed to test the knowledge gap hypotheses and to examine whether awareness was associated with physical activity related beliefs (i.e., outcome expectations, self-efficacy), intention, and LTPA. All analyses included those respondents who were 20 years of age and older in 2007/2008 (N = 4424).

**Results:**

Approximately 8% of Canadians were still aware of ParticipACTION unprompted and 82% were aware when prompted. Both education and income were significant correlates of awareness among Canadians. The odds of people being aware of ParticipACTION were greater if they were more educated and reported higher income. Awareness of ParticipACTION was also associated with outcome expectations, self-efficacy, intention, and LTPA status.

**Conclusion:**

Awareness of ParticipACTION is associated with LTPA. Knowledge gaps in awareness are associated with level of education and household income. Thus, future promotion campaigns should include specific strategies to target different segments of the population, especially people who are living in deprived conditions with lower levels of education.

## Background

While leisure-time physical activity has increased in Canada over the past 20 years [1], approximately 50% of the Canadian population does not achieve the recommended minimum physical activity [2]. Physical inactivity thus constitutes a major public health concern [3] with related social and economic costs [4]. Mass media campaigns are an initial strategy for increasing awareness about physical activity at a population level [5-8]. Though mass media interventions alone are not expected to be effective for increasing physical activity [9], evidence exists to support the role of media campaigns in changing beliefs and knowledge about physical activity [6,10]. In light of this, identifying and examining both the potential contributions and limitations of such interventions is required to fully determine the role of media campaigns for increasing physical activity. Thus, a further understanding of communication or media campaigns to promote physical activity would make an important contribution to the literature and assist the inception of campaigns elsewhere.

ParticipACTION was an innovative and pervasive communication campaign and integrated social marketing strategy [11] that promoted physical activity in the Canadian population over three decades. Built on the principles of social marketing and health communication, ParticipACTION aimed to increase awareness of the benefits of active living and develop supportive environments for physical activity [12]. ParticipACTION's campaigns were characterized by creative marketing techniques, strategic partnerships and emphasis on community mobilization [12,13]. Launched in 1971 with seed money from a federal government concerned by rising health care costs and declining health and fitness standards, the ParticipACTION campaign evolved over 29 years before its official conclusion in 2000. Its stated purpose was two-fold: to motivate all Canadians to be more active and to improve population levels of fitness over the long term [12].

Formal evaluation techniques designed to appraise public health programs were limited at the time of ParticipACTION's inception [14]. Therefore, specific logic models were not implemented to either design or evaluate the campaign. However some evidence exists to suggest significant population awareness and support of ParticipACTION. A review by Bauman et al. [14] synthesized results from six national surveys and revealed high levels of unprompted and prompted recall of ParticipACTION among Canadian adults over the course of two decades. When asked to list fitness and physical activity organizations (unprompted recall) ParticipACTION was consistently mentioned by between one-sixth and one-third of Canadians between 1978 and 1994. Prompted recognition rates of the ParticipACTION logo or messages ranged from 77% in 1977 to over 80% from 1980 through to 2002. Of those aware of the organization, 83% to 95% thought it useful in promoting physical activity participation. A review of physical activity campaigns around the world suggested that prompted recall rates of campaigns, logos or messages post-campaign were on average 70% from population surveys [10]. The prompted recall rates of 80% across ParticipACTION surveys indicate a persistent and pervasive impact on knowledge and awareness.

According to the hierarchy-of-effects model (HOEM) [10,15] awareness of campaigns should influence behaviour by changing targeted intermediate mediators such as beliefs (e.g., outcome expectations and self-efficacy) and intentions for the behaviour among the target group. Thus, evaluations of mass media campaigns should first establish if there are proximal (immediate) effects, such as awareness of the campaign and understanding of the message on the part of individuals, prior to evaluating the more distal goals of perceived relevance, changes in beliefs or intentions, and ultimately behaviour change. In evaluating awareness it is important to first measure unprompted awareness (e.g., what are important sources of physical activity information?) followed by prompted awareness (e.g., are you aware of ParticipACTION?). Awareness, understanding, and perceptions of an initiative and its messages, and the formation of social norms are examples often the focus of mass media campaign evaluations [5]. During ParticipACTION's tenure increases in physical activity levels were observed among Canadians between 1981 and 1995 [1,6]. However, these changes cannot be uniquely linked to ParticipACTION as other policy, environmental and public health factors may have also played a role.

A recent survey suggests that ParticipACTION has maintained its presence in the vocabulary of Canadian adults despite the lack of new national public service messages since 1999 and the fact that it ceased all operations in 2001 [16]. The survey assessed awareness of ParticipACTION, its taglines and usefulness using the Canadian Fitness and Lifestyle Research Institute's Physical Activity Monitor (PAM). The study, which was conducted from November 2004 through to January 2005, determined the degree of population attention to and recall of ParticipACTION and its perceived usefulness among adult Canadians. Participants were asked questions such as "When you think of physical fitness, what group or organization promoting fitness in Canada comes to mind?" (unaided recall) and "Have you ever heard of ParticipACTION?" (prompted recall). In response to the unaided recall question, 15% cited ParticipACTION, third behind private exercise clubs and organizations (29%) and public facilities such as YMCA's (20%). More than three quarters of Canadians (78%) indicated they had heard of ParticipACTION when prompted. Furthermore, the majority of adults who were aware of ParticipACTION considered its efforts to be either somewhat useful (47%) or very useful (29%) in promoting physical activity. In general, Canadians with higher levels of education and income were more likely to report hearing of ParticipACTION and more likely to cite it when answering the unaided recall question. Adults between the ages of 25-64 demonstrated greater awareness of ParticipACTION than those 18 years of age or younger.

While these findings suggest the ParticipACTION campaign was recognized by the majority of the Canadian adult population, they are also evidence of the knowledge gap hypothesis [17] in which it is proposed that people of higher socioeconomic status (SES) benefit more from information flowing into a social system (e.g., through media campaigns) than do people of lower SES. Thus, public health campaigns and initiatives such as ParticipACTION may actually exacerbate existing differences between SES groups in both behaviour and health. Though empirical research has confirmed the existence of the knowledge gap for many phenomena [18,19] little is known about the origin of this gap or how the gap develops in time in relation to physical activity campaigns.

Much evidence exists to support the claim that beliefs such as the outcomes people expect from being physically active (i.e., outcome expectations) and confidence in their ability to be physically active (i.e., self-efficacy) are strong correlates of physical activity [20,21]; however, it is less clear whether these constructs can be influenced by media campaigns and then subsequently influence physical activity as suggested by the HOEM, especially in the Canadian context. If awareness is influenced by SES and associated with motivational factors, then these are potentially important to consider in the logic model(s) for developing and evaluating any new campaigns. More importantly, if the campaign itself contributes to gaps in knowledge then specific dissemination strategies should be developed to target those groups not receiving the knowledge. Therefore, the purposes of this study were to (a) determine the current awareness of ParticipACTION among Canadians; (b) confirm if awareness of the ParticipACTION initiative among Canadians varied as a function of education and income levels; and, (c) to examine whether awareness of ParticipACTION was associated with physical activity related beliefs, intentions, and physical activity as suggested by the HOEM. Specifically, we tested a model including awareness of ParticipACTION (unprompted, prompted), outcome expectations, self-efficacy, intention, and physical activity status. We hypothesized people who were aware of ParticipACTION would be more likely to be physically active and to hold more positive beliefs about being physically active.

## Methods

### Participants

To assess awareness of ParticipACTION on the part of Canadians, a population-based survey was conducted on a monthly basis over a period of 6 months from mid-August 2007 to February, 2008. The survey consisted of a set of additional questions on the 2007 Physical Activity Monitor (PAM) conducted by the Institute for Social Research (ISR) at York University on behalf of the Canadian Fitness and Lifestyle Research Institute (CFLRI). The sample was selected on a probability basis proportional to the size of the population in the provinces and territories, with an over-sample in small provinces. For the 2007 PAM, 333 Canadians, 15 years of age and older, were cross-sectionally sampled per month across all provinces and territories except Nunavut. This on-going rolling sampling process yielded a nationally representative sample. To allow for more in-depth comparisons (e.g., regional and demographic), an additional 470 Canadians on average were sampled per month resulting in a total monthly sample of about 800. Therefore 4,650 Canadians were initially involved in the survey on ParticipACTION with a response rate of 49%. Because the ParticipACTION campaign ceased in 1999, the sample was limited to those respondents who were 20 years of age and older in 2007/2008. Therefore, our final sample included 4424 respondents.

In the past, the response rate for the PAM has been approximately 50% [1]. This is comparable to other surveys conducted by the ISR and exceeds that of many other surveys (e.g., Garcia Bengoechea et al. [22]). Non-response has not been related to the particular subject matter and non-response bias has been minimal since prevalence rates for key variables have not varied between those responding and those converted from initial refusals (p = 0.9) [1].

### Measures

This study is part of a larger ongoing evaluation of ParticipACTION communications in which physical activity, beliefs about physical activity (e.g., outcome expectations, self-efficacy, intentions), perceptions of the built environment, and awareness of ParticipACTION among Canadians are being measured. For the purposes of this study, we will describe and report on the awareness, beliefs, and physical activity variables.

#### Demographics

Information was collected on participants' sex, age, household income, and level of education (high school or less, college, university).

#### Physical Activity Beliefs

Outcome expectations were measured with three items asking about beliefs that regular physical activity will help reduce stress, prevent heart disease, and maintain activities of daily living. On a 7-point scale, response options ranged from 'do not agree' to 'agree very strongly'. These items were then aggregated to create a mean score (Cronbach's alpha = .70). Self-efficacy was measured with a single question asking "how confident are you that you can regularly do a total of 30 minutes or more of moderate physical activity per day at least three or four times a week?" On a 5-point scale, response options ranged from 'not at all confident' to 'very confident'. Intention to be physically active was measured with a single question asking "thinking ahead over the next six months, to what extent do you intend to be physically active?" On a 7-point scale, response options ranged from 'no intention at all' to 'fully intend'.

#### Awareness of ParticipACTION

Two categories of ParticipACTION awareness were assessed: unprompted awareness and prompted awareness. First, for unprompted awareness, participants were asked "When you think of physical fitness, what group or organization promoting fitness in Canada comes to mind?" For prompted awareness, participants were then asked if they were aware of the following programs and/or campaigns as potential sources of physical activity information including *Canada's Food Guide to Health Eating*, *Body Break*, *Canada on the Move*, *Canada's Physical Activity Guide to Health Active Living*, and ParticipACTION. A similar methodology was adopted in a previous evaluation of ParticipACTION [16] and by Craig et al. [23,24] to assess the impact of the *Canada on the Move *initiative.

#### Physical Activity

Leisure time physical activity (LTPA) over the past 12 months was assessed using an adaptation of the Minnesota Leisure Time Physical Activity Questionnaire [25]. Participants indicated which activities they had undertaken in the previous year (up to 22 activities), and the frequency, and the average time spent in each. Average daily LTPA was calculated as follows: LTPA (MET hours) = Σ (N_i × D_i × METs_i/365) where N is the number of times the activity was performed in the past 12 months, D is the average duration in hours, and METs is the estimated energy cost (kJ×kg^-1 ×hr^-1). Respondents were classified as being sufficiently active if they achieved 1.5 MET-hours or more per day of LTPA [1] which is roughly equivalent to accumulating 30 minutes of moderate physical activity on most days of the week.

### Procedures

Individuals were selected using the PAM/ISR two-step-design beginning with a random digit dialling protocol to select an eligible household telephone number. The individual, 15 years of age and older, with the closest birth date was then selected. Once a potential respondent within a household was chosen, no other person within that same household could participate in the survey.

Interviews were administered by the ISR using Computer Assisted Telephone Interviewing (CATI) technology. Respondents were interviewed in either French or English. The CATI system was programmed to check responses for appropriateness of range and logical consistency at the time of data entry. According to the ISR, this approach substantiated reliability and validity for telephone interviewing protocols.

The PAM was reviewed by a departmental Research Ethics Board (REB) at York University. For the purposes of this project, the survey also received ethics review at the University of Alberta.

### Analysis

Analyses were performed using SPSS 16. Frequencies were calculated for the awareness variables and associations between the sociodemographic variables (including LTPA status) and awareness were determined by calculating chi square statistics with significance assessed at p < 0.05 (**purposes a and b**). Two binary logistic regression models were then constructed to test the knowledge gap model in which awareness of ParticipACTION (unprompted or prompted) was regressed on education and income, while controlling for gender and age (**purpose b**). To test the HOEM (**purpose c**), we first conducted a series of one-way between-subjects ANCOVAs for outcome expectations, self-efficacy, and intention with type of awareness for ParticipACTION (unprompted, prompted) as the independent variable and gender and age as covariates. Then LTPA status (active, inactive) was regressed on the beliefs and awareness variables in two hierarchical logistic regressions. Specifically, age, gender, education, income, and awareness of ParticipACTION (unprompted or unprompted) were entered on the first step, followed by outcome expectations, self-efficacy on step 2, with intention entered on the last step. Finally, all covariates in the analyses were centered to reduce the possibility of multicollinearity [26] and all analyses were weighted to reflect the sample design, and age and gender distribution of Canadians.

## Results

Frequencies of the sociodemographic characteristics along with their unadjusted associations with ParticipACTION awareness are presented in Table 1. Approximately 56% of respondents were categorized as being sufficiently active, 8% were aware of ParticipACTION unprompted in that they cited it as the organization they identified when thinking of physical activity, while 82% were aware when prompted. When prompted about other programs and campaigns, 90% of Canadians were aware of Canada's Food Guide to Healthy Eating, while 67%, 34%, and 22% indicated they were aware of Body Break, Canada's Physical Activity Guide to Healthy Active Living, and Canada on the Move respectively.

**Table 1:** Associations between Sociodemographic Factors and Awareness of ParticipACTION among Canadian Adults (weighted percentages)

			Awareness of ParticipACTION					
	
	Total Sample		Prompted awareness			Unprompted awareness		
	n	%	*n*	%	p	*n*	%	p
**Overall**	4424	---	3800	81.6		389	8.2	
**Gender**								****
Male	1923	48.2	1670	82.6		210	12.2	
Female	2501	51.8	2130	80.6		179	6.7	
**Age (years)**					****			****
20-24	230	7.3	130	46.6		4	0.8	
25-44	1707	41.5	1466	82.2		154	10.2	
45-64	1811	33.4	1657	90.0		206	12.6	
65+	676	17.7	547	78.6		25	4.4	
**Household income ($)**					****			****
< 30,000	723	16.0	558	67.9		28	3.3	
30,00-59,999	1135	30.4	970	80.6		83	7.2	
60,000+	1794	53.5	1634	87.9		212	12.8	
**Level of education**					****			****
High school or less	1557	35.2	1261	75.8		80	6.4	
College	1355	30.7	1200	86.0		122	8.9	
University	1445	34.1	1290	84.2		183	13.0	
**LTPA status**					****			*
Inactive	1932	44.1	1591	77.1		139	7.5	
Active	2488	55.9	2205	85.1		250	10.9	

LTPA = leisure time physical activity.

* p < .05

*** p < .001.

**** p < .0001.

Higher awareness of ParticipACTION was reported by men (12%) than women (7%) unprompted. No gender differences, however, existed for prompted awareness. The youngest group of respondents were much less likely to be aware of the organization regardless of the awareness condition. If respondents were deemed to be sufficiently active they were more likely to be aware of ParticipACTION. Finally, similar patterns of awareness were observed for both household income and level of education with those at the lowest levels being less aware of ParticipACTION than those at higher levels. These patterns occurred for both types of awareness and support the idea of a knowledge gap. After controlling for age and gender, both education (OR = 1.31, 95% CI 1.11-1.55; OR = 1.13, 95% CI 1.00-1.28) and income (OR = 1.75, 95% CI 1.40-2.19; OR = 1.94, 95% CI 1.71-2.22) were significantly associated with unprompted and prompted awareness of ParticipACTION respectively.

As a first step in examining the utility of the HOEM in relation to LTPA status, we conducted a series of one-way ANCOVAs to determine if outcome expectations, self-efficacy, and intention for physical activity varied with level of awareness for ParticipACTION. After adjustment by covariates, outcome expectations (F [1, 2527] = 6.01, p = .014, partial eta squared = .002), self-efficacy (F [1, 2513] = 7.89, p = .005, partial eta squared = .003), and intention (F [1, 2504] = 9.32, p = .002, partial eta squared = .004), varied significantly with unprompted awareness of ParticipACTION. Similarly, outcome expectations (F [1, 2531] = 92.62, p < .0001, partial eta squared = .04), self-efficacy (F [1, 2517] = 46.42, p < .0001, partial eta squared = .02), and intention (F [1, 2508] = 27.29, p < .0001, partial eta squared = .01), varied significantly with prompted awareness of ParticipACTION. The adjusted marginal means, as displayed in Table 2, show that higher levels of outcome expectations, self-efficacy, and intention for physical activity were held by those who were aware of ParticipACTION regardless of the type of awareness.

**Table 2:** Estimated Marginal Means (standard error)^a ^for Physical Activity Related Outcome Expectations, Self-efficacy, and Intention by Type of Awareness for ParticipACTION

	Unprompted Awareness		Prompted Awareness	
	
	Unaware	Aware	Unaware	Aware
Outcome expectations	6.51 (0.02)	6.64 (0.05)	6.22 (0.04)	6.59 (0.02)
Self-efficacy	4.00 (0.03)	4.24 (0.08)	3.67 (0.06)	4.10 (0.03)
Intention	5.93 (0.3)	6.22 (0.09)	5.67 (0.06)	6.03 (0.03)

^a ^Adjusted for gender and age.

When LTPA status was regressed on the beliefs and awareness variables associated with the HOEM (see Table 3), both self-efficacy and intention were significant covariates regardless of the awareness condition. Awareness was a significant covariate of LTPA status too; however the association appeared to be stronger and more consistent in the prompted condition. In that case, if a person was aware of ParticipACTION, the odds of he/she being physical active increased by a factor of 1.53 to 1.81 depending on whether the beliefs were included in the model or not. Given the reductions in size of both the Wald statistic and beta at step 2 for both awareness conditions, it appears outcome expectations and self-efficacy accounted for some of the association between awareness and LTPA. Outcome expectations were associated with LTPA status for the unprompted condition only.

**Table 3:** Adjusted and Weighted Odds^a ^Ratios for Factors Associated with Physical Activity (LTPA) Status by both Prompted and Unprompted Awareness of ParticipACTION

	Step 1				Step 2				Step 3			
	b	Wald	OR	95%CI	b	Wald	OR	95%CI	b	Wald	OR	95%CI
Unprompted Awareness												
Awareness	0.40	5.48	1.49	1.07-2.08*	0.31	3.20	1.37	0.97-1.93	0.29	2.59	1.33	0.94-1.88
Outcome expectations					0.13	3.84	1.14	1.01-1.31*	0.09	1.61	1.09	0.95-1.25
Self-efficacy					0.55	168.87	1.73	1.59-1.88****	0.43	88.48	1.54	1.41-1.69****
Intention									0.27	35.46	1.31	1.20-1.43****
Prompted Awareness												
Awareness	0.56	19.40	1.74	1.36-2.23****	0.39	8.55	1.48	1.14-1.92**	0.39	8.68	1.49	1.14-1.94**
Outcome expectations					0.12	2.67	1.11	0.97-1.28	0.06	0.85	1.07	0.93-1.23
Self-efficacy					0.55	166.80	1.72	1.59-1.87****	0.43	86.79	1.54	1.40-1.68****
Intention									0.27	35.74	1.31	1.20-1.43****

^a^Adjusted for gender, age, education, and income.

* p < .05

** p < .01

**** p < .0001

## Discussion

ParticipACTION was a social marketing and communications organization that promoted physical activity in Canada for approximately 30 years before its official conclusion in 2001. We found that approximately 8% of Canadians were still aware of ParticipACTION unprompted and 82% were aware when prompted. In comparison to rates of unprompted awareness of 30% in 1989 and 17% in 1994 [14] and 15% in 2004 [15], this reflects a definite decay in "top of mind" awareness of ParticipACTION between 1994 and 2007. This is also reflected by the fact that middle-aged and older Canadians were more aware of ParticipACTION than younger Canadians. Even when prompted, only 47% of people between the ages of 20-24 years were aware of the organization compared to 90% awareness among those 45-64 years of age.

The second purpose of this study was to determine if knowledge gaps in awareness of ParticipACTION existed and if these gaps were differentially related to household education and perhaps income. Both education and income were significant correlates of awareness among Canadians. The odds of people being aware of ParticipACTION were greater if they were more educated and reported higher income. These findings are consistent with the deficit model of knowledge gap phenomena [27] which suggests that the higher the education of an individual, the higher his/her motivation to attend to and comply with health messages.

The final purpose of this study was to determine if awareness of ParticipACTION was associated with physical activity related beliefs, intentions, and physical activity. We found good support for the use of HOEM [15] in that outcome expectations, self-efficacy, and intention varied significantly by awareness regardless of the awareness condition (unprompted, prompted). However, the sizes of the effects in the ANCOVAs were very small suggesting that other factors are influencing the beliefs and intention for physical activity apart from awareness. We also found that awareness of ParticipACTION was associated with LTPA status. However, this association was more apparent for the prompted condition. Consistent with the HOEM and other social cognitive models [28,29], self-efficacy and intention were strong correlates of LTPA status. In fact, much of the association between awareness and LTPA status appeared to be attenuated with the addition of these constructs into the model for both awareness conditions. Thus the relationship between awareness and physical activity may be mediated by beliefs about physical activity. However, given the cross-sectional nature of the current data, this observation can only be verified in a longitudinal design (e.g., Bauman et al. [30]). We are not certain why awareness of ParticipACTION was independently associated with LTPA status in the prompted condition but not in the unprompted condition. By prompting people, it is possible that ParticipACTION was made a more salient behavioural target for people. Alternatively, it is possible that some individuals who were aware of ParticipACTION prior to its demise in 2001 were also aware of other organizations that continued to be in operation over the period (e.g. fitness studios like Curves, Heart and Stroke Foundation). In that case the other organizations may be more salient to those respondents at the time of the survey, which could influence their propensity to recall ParticipACTION relative to others without prompting, thereby attenuating the relationship between unprompted recall of ParticipACTION and current LTPA.

ParticipACTION was recently re-launched thus presenting a unique opportunity to better understand its potential to influence beliefs, intentions and behaviour through strategic evaluation [31]. Our findings have both theoretical and practical importance for the new campaign. First, the HOEM is a useful theoretical framework for evaluating the mechanisms of influence of media campaigns on population-level physical activity and related beliefs. It appears that awareness of such campaigns is a central initial indicator of campaign effectiveness. But it was also apparent that, while awareness and knowledge of ParticipACTION and its messages on the part of the Canadian population is necessary, it is not sufficient if the campaign is to be effective in changing beliefs and behaviour [5,6]. Second, education and income are critical determinants of knowledge about physical activity messages. Thus, levels of education and income are important factors to consider when designing and investigating population-based health promotion campaigns for physical activity. In particular, theoretical-based interventions that include information campaigns to influence knowledge and beliefs about physical activity should be tailored to be more salient for the intended audience [30]. Tichenor and colleagues [17] identified communication skills, prior knowledge, relevant social contact, selective exposure and information storage, and the nature of the mass media system as explanations for education-related knowledge gaps. Thus, people with higher levels of education are more likely to expose themselves to mass media (e.g., read the newspaper), comprehend the information provided, and then have the social networks within which the messages are reinforced and valued. Therefore, attempts to lessen the knowledge gap about ParticipACTION could target specific segments of the population [32] and take into consideration these underlying processes.

In the past, the more information-rich media was consumed by better educated people while television was the source of information for the less educated segment of the population [33]. Bonfadelli [34] suggests that knowledge gaps that occur in relation to 'old media' such as television and print news appear to stem largely from differences in interest to the topic. However, when considering the knowledge gap and internet use, there are new factors to consider. For example, compared to traditional media forms, the supply of information is conceivably unlimited on the internet and is more heterogeneous than the information journalists have access to. Additionally, internet use requires more technical savvy and is more dependent on access than old media. Therefore, the internet could act to promote individualized information seeking and increase heterogeneity within the audience [34]. Data from two Swiss studies [34] found that internet use was associated with education level, income, sex and age. The author also found that diffusion and adoption of internet took place earlier and faster in high SES groups than in lower SES groups. Gaps in the usage of internet were related to variations in internet access and variations in content specific use. Gaps in the way the internet was used were primarily based on education. For example, people with higher education used the internet more for information seeking while people with lower levels of education used the internet significantly more for entertainment purposes. Therefore, physical activity promotion campaigns that use the internet will not be immune to the knowledge gap. If future ParticipACTION campaigns are planned for the internet, then strategies need to be put into place to overcome the potential knowledge gap phenomenon.

Strengths of the study include the validated survey and sampling methodology from an existing surveillance system (i.e., PAM) and the application of theoretical frameworks to guide the selection of questions and data analysis. However, this study is not without limitations that should be acknowledged. The use of single-item measures for self-efficacy, outcome expectations, intention, and the awareness variables, along with the lower response rate, may limit the reliability of our findings. As mentioned previously, the cross-sectional design prevents any discussion of cause and effect and limits the formal testing of the hierarchy of effects in the HOEM. While we were able to control for some demographic factors that may have accounted for the observed associations, other variables such as perceived health status and immigrant status may have been confounders for associations between intention and, or, LTPA and awareness. For instance, recent immigrants to Canada may be unaware of ParticipACTION regardless of their intentions to be physically active and levels of education or income. Exposure to media and media consumption are important variables to consider with regard to the effectiveness of a media campaign and the knowledge gap. Unfortunately, we had no such measures in our study. Finally, Kang [19] suggests that both awareness knowledge (i.e., the existence of the campaign) and depth of knowledge (i.e., actual message and contents) should be assessed when evaluating health media campaigns. With our items, we measured awareness knowledge but not depth knowledge. Future studies on the topic are advised to measure both types of knowledge while exploring the context in which campaign messages are received, interpreted, and used by audiences [35].

In summary, Canadians remain aware of ParticipACTION even though its campaigns have not been running for approximately 10 years. Those Canadians who are aware of ParticipACTION are more likely to hold positive beliefs about physical activity and to be physically active. However, a definite decay in awareness has occurred and younger adults are less likely to be aware of the campaign even when prompted. Knowledge gaps in awareness are associated with levels of education and household income. Thus, future physical activity promotion campaigns (i.e., the new ParticipACTION) need specific strategies to target different segments of the population, especially people who are living in deprived conditions with lower levels of education. Otherwise, such campaigns will actually increase the knowledge gap between these groups resulting in less opportunity to take advantage of programs and information [27].

## Competing interests

Four of the authors (CLC, LRB, JCS, MST) have been involved on various committees and advisory groups for both the previous and 'new' ParticipACTION including the Board of Directors (MST). The Canadian Fitness and Lifestyle Research Institute (CFLRI) is contracted by the 'new' ParticipACTION to perform ongoing research and surveillance - CLC is President of CFLRI but receives no personal compensation for the contracted work. This project was completed before the contract between ParticipACTION and CFLRI was in place.

## Authors' contributions

JCS conceived of the study, participated in its design, conducted the statistical analysis, and drafted the manuscript. MT and LB conceived of the study and contributed to the manuscript. CC oversaw the data collection and provided input on the manuscript. AB, GF, KC, and RP helped to draft the manuscript and contributed to the grant proposal that funded the study. MC assisted with drafting the manuscript. All authors read and approved the final manuscript.
